# Association between Noninvasive Fibrosis Markers and Chronic Kidney Disease among Adults with Nonalcoholic Fatty Liver Disease

**DOI:** 10.1371/journal.pone.0088569

**Published:** 2014-02-10

**Authors:** Giorgio Sesti, Teresa Vanessa Fiorentino, Franco Arturi, Maria Perticone, Angela Sciacqua, Francesco Perticone

**Affiliations:** Department of Medical and Surgical Sciences, University “Magna Graecia” of Catanzaro, Catanzaro, Italy; Consiglio Nazionale delle Ricerche, Italy

## Abstract

Evidence suggests that nonalcoholic fatty liver disease (NAFLD) and non-alcoholic steatohepatitis (NASH) are associated with an increased risk of chronic kidney disease (CKD). In this study we aimed to evaluate whether the severity of liver fibrosis estimated by NAFLD fibrosis score is associated with higher prevalence of CKD in individuals with NAFLD. To this end NAFLD fibrosis score and estimated glomerular filtration rate (eGFR) were assessed in 570 White individuals with ultrasonography-diagnosed NAFLD. As compared with subjects at low probability of liver fibrosis, individuals at high and intermediate probability showed an unfavorable cardio-metabolic risk profile having significantly higher values of waist circumference, insulin resistance, high sensitivity C-reactive protein, fibrinogen, uric acid and lower insulin-like growth factor-1 levels. Individuals at high and intermediate probability of liver fibrosis have lower eGFR after adjustment for gender, smoking, glucose tolerance status, homeostasis model assessment index of insulin resistance (HOMA-IR index), diagnosis of metabolic syndrome, statin therapy, anti-diabetes and anti-hypertensive treatments (*P* = 0.001). Individuals at high probability of liver fibrosis had a 5.1-fold increased risk of having CKD (OR 5.13, 95%CI 1.13–23.28; *P* = 0.03) as compared with individuals at low probability after adjustment for age, gender, and BMI. After adjustment for glucose tolerance status, statin therapy, and anti-hypertensive treatment in addition to gender, individuals at high probability of liver fibrosis had a 3.9-fold increased risk of CKD (OR 3.94, 95%CI 1.11–14.05; *P* = 0.03) as compared with individuals at low probability. In conclusion, advanced liver fibrosis, determined by noninvasive fibrosis markers, is associated with CKD independently from other known factors.

## Introduction

Renal dysfunction is a worldwide health problem as a consequence of its adverse outcomes including cardiovascular events and all-cause-mortality [1]–[3]. Increasing evidence suggests that chronic kidney disease (CKD), defined as a sustained reduction in the glomerular filtration rate (GFR), and cardiovascular disease share common risk factors such as the metabolic syndrome and its individual components (elevated blood pressure, high plasma glucose levels, high triglycerides levels, low high density lipoprotein (HDL) levels, and abdominal obesity) [4]–[6].

Nonalcoholic fatty liver disease (NAFLD) is the most common chronic liver disease in Western countries [7], comprising a large spectrum of disorders ranging from simple steatosis, to non-alcoholic steatohepatitis (NASH) with increasing levels of fibrosis, and, ultimately, cirrhosis [8], [9]. While simple liver steatosis is considered as a non-progressive condition, NASH is a potentially harmful disorder associated with increased risk of liver-related morbidity and mortality [10]–[13]. Like CKD, NAFLD has been linked to the same cluster of cardio-metabolic risk factors including the metabolic syndrome and its individual components [14], [15], and is associated with increased risk of incident cardiovascular events [11]–[13], [16], [17]. Increasing evidence suggests that NAFLD/NASH and CKD share several traditional and non-traditional cardio-metabolic risk factors including higher plasma inflammatory and hemostatic factors, hyperuricemia, lower circulating insulin-like growth factor-1 (IGF-1) levels, endothelial dysfunction, and oxidative stress biomarkers [17]–[26]. Additionally, a number of studies has shown that liver biopsy–proven or ultrasonography-diagnosed NAFLD in adult subjects is associated with an increased prevalence [26]–[29] and incidence [30], [31] of CKD. Interestingly, studies that used liver biopsy to diagnose NAFLD have shown that the severity of NASH histology (i.e., fibrosis stage) is associated with a more pronounced kidney dysfunction, independently of potential confounding factors, including the metabolic syndrome and its individual components [28], [29]. Liver biopsy is considered the gold standard for the assessment of hepatic fibrosis and inflammation severity in subjects with chronic liver disease but has a number of limitations, including sampling variability, invasiveness, complications, and costs [32] making it unfeasible for large epidemiological studies. In an attempt to overcome these problems, several noninvasive scoring indexes have been developed by combining clinical and biochemical parameters that are useful to categorize subjects with NAFLD in subgroups at low and high risk of advanced fibrosis [33]–[37]. Recent studies have shown that advanced fibrosis, as determined by the noninvasive NAFLD fibrosis score [34], is a significant predictor of mortality, mainly from cardiovascular causes, in individuals with ultrasonography-diagnosed NAFLD [38], [39]. The clinical utility of NAFLD fibrosis score in assessing CKD in individuals with ultrasonography-diagnosed NAFLD is still unsettled. In the present study, we therefore determined whether the severity of liver fibrosis estimated by the NAFLD fibrosis score is associated with higher prevalence of CKD among individuals with ultrasonography-diagnosed NAFLD.

## Materials and Methods

The study group comprised 570 White individuals participating to the CATAnzaro MEtabolic RIsk factors (CATAMERI) study, a cross-sectional study assessing cardio-metabolic risk factors in individuals carrying at least one risk factor including dysglycemia, overweight/obesity, hypertension, dyslipidemia, and family history for diabetes [40]. Information regarding medical history, drug use, alcohol, and cigarette consumption were collected. Exclusion criteria included: history of malignant disease, liver cirrhosis, gout, chronic gastrointestinal diseases, chronic pancreatitis, regular use of steatosis-inducing drugs, self-reporting alcohol intake of 3 or more drinks per day, positivity for antibodies to hepatitis C virus (HCV) or hepatitis B surface antigen (HBsAg). Clinical cardiovascular disease was excluded on the basis of medical history and resting electrocardiogram.

All anthropometric and serological measurements were made in the morning after a 12-h fasting using standardized methods. Brachial blood pressure was measured in the left arm of the supine subjects, after 5 min of quiet rest, with a digital electronic tensiometer (regular or large adult cuffs were used according to arm circumference). A minimum of three blood pressure readings were taken on three separate occasions at least 2 weeks apart, and the medians of these three values were used. A 75 g oral glucose tolerance test (OGTT) was performed with sampling for plasma glucose.

Liver ultrasonography was performed in all participants by the same trained operator, who was blinded to participants' details, using a Toshiba Aplio 50 ultrasound apparatus equipped with a 3.5-MHz linear transducer [20], [21]. Longitudinal, sub costal, ascending, and oblique scans were performed. The ultrasonographic criteria used to diagnose fatty liver included liver and kidney echo discrepancy, the presence of an increased liver echogenicity or “bright liver”, poor echo penetration into the deep portion of the liver, and vascular blurring either singly or in combination.

The protocol was approved by the local ethical committee (Comitato Etico Azienda Ospedaliera “Mater Domini”, Catanzaro, Italy), and written informed consent was obtained from all participants in accordance with principles of Helsinki Declaration.

### Analytical determinations

Glucose, triglycerides, total and HDL cholesterol concentrations were determined by enzymatic methods (Roche, Basel, Switzerland). Alanine aminotransferase (ALT) and aspartate aminotransferase (AST) levels were measured using the α-ketoglutarate reaction; gamma-glutamyltransferase (GGT) levels with the L-gamma-glutamyl-3-carboxy-4-nitroanilide rate method. Serum creatinine was measured in the routine laboratory by an automated technique based on a Creatinine Jaffè compensated method for serum and plasma (Roche Diagnostics) implemented in an auto-analyzer. Serum uric acid was measured by the URICASE/POD method implemented in an auto-analyzer (Boehringer Mannheim, Mannheim, Germany). Albumin concentration was determined with a Alb2 kit on a Cobas C6000 analyzer (Roche Diagnostics, Milan, Italy). High sensitivity C-reactive protein (hsCRP) levels were measured by automated instrument (CardioPhase® hsCRP, Milan, Italy). An automated nephelometric technology using the BN™ II System analyzer (Siemens Healthcare, Italy) was employed to measure plasma fibrinogen concentrations. Plasma insulin concentration was measured with a chemiluminescence-based assay (Immulite®, Siemens, Italy), and total serum IGF-1 concentrations were determined by chemiluminescent immunoassay (Nichols Institute Diagnostic, San Juan Capistrano, CA).

### Definitions

Glucose tolerance status was diagnosed according to the American Diabetes Association (ADA) criteria [41]: normal glucose tolerance (NGT) when fasting plasma glucose (FPG) was <5.6 mmol/l and 2 h post-load <7.8 mmol/l, isolated impaired fasting glucose (IFG) when FPG was 5.6–6.9 mmol/l and 2 h post-load <7.8 mmol/l, impaired glucose tolerance (IGT) when FPG was ≤6.9 mmol/l and 2-h post-load was 7.8–11.0 mmol/l, type 2 diabetes when FPG was ≥7.0 mmol/l and/or 2 h post-load was ≥11.1 mmol/l.

The NAFLD fibrosis score was calculated according to the following formula: −1.675 + 0.0373 x age + 0.0943 x BMI +1.13 x IFG or diabetes (yes = 1, no = 0) + 0.99 x AST/ALT ratio −0.013 x platelet − 0.66 x albumin [20]. Two cutoff points (>0.676 and <−1.455) were used to divide the subjects in three groups: low risk of fibrosis (< −1.455), intermediate risk of fibrosis (−1.455–0.676), and high risk of fibrosis (>0.676) [34].

The homeostasis model assessment index of insulin resistance (HOMA-IR) was calculated as fasting insulin × fasting glucose/22.5 [42].

Estimated glomerular filtration rate (eGFR) was calculated by using the CKD-EPI equation [43]: eGFR =  141 x min(Scr/k, 1)^α^ x max(Scr/k, 1)^−1.209^ x 0.993^Age^ x 1.018 [if female], where Scr is serum creatinine, k is 0.7 for females and 0.9 for males, α is −0.329 for females and −0.411 for males, min indicates the minimum of Scr/k or 1, and max indicates the maximum of Scr/k or 1. CKD was defined as eGFR <60 ml/min/1.73 m^2^.

Metabolic syndrome was defined as having three or more of the following criteria [44]: waist circumference >102 cm in men and >88 cm in women, triglycerides >1.69 mmol/l or on treatment for elevated triglycerides, HDL <1.04 mmol/l in men and <1.3 mmol/l in women or on treatment for reduced HDL, blood pressure >130/85 mmHg or on antihypertensive treatment, fasting glucose ≥5.6 mmol/l.

### Statistical analysis

Variables with skewed distribution including triglycerides, hsCRP, GGT, and fasting insulin were natural log transformed for statistical analyses. Continuous data are expressed as means ± SD. Categorical variables were compared by χ2 test. Anthropometric and metabolic differences between groups were tested after adjusting for gender using a general linear model with *post hoc* Bonferroni correction for multiple comparisons. To avoid overestimation of the model, we excluded those variables used as a part of the NAFLD fibrosis score calculation i.e. age, and BMI. A general linear model was used to determine the independent impact on eGFR values of several variables including smoking habit, glucose tolerance status, HOMA-IR index, diagnosis of metabolic syndrome, statin therapy, medications for diabetes, anti-hypertensive treatments, and gender.

A logistic regression analysis adjusted for gender, age, and BMI was used to determine the association between the study groups and CKD. A second logistic regression model adjusted for glucose tolerance status, statin therapy, and anti-hypertensive treatment in addition to gender was run to determine the association between the study groups and CKD. A *P* value <0.05 was considered statistically significant. All analyses were performed using SPSS software program Version 16.0 for Windows.

## Results

The clinical and biochemical features of the study group are reported in Table 1. The mean age for the entire cohort was 54.1±13.5 yrs. Of the 570 subjects examined, 220 (38.6%) had NGT, 63 (11.1%) had IFG, 112 (19.6%) had IGT, and 175 (30.7%) had type 2 diabetes. Metabolic syndrome was diagnosed in 392 (68.8%) individuals, and 368 (64.6%) subjects had hypertension treated with anti-hypertensive medications. A low probability of advanced liver fibrosis (NAFLD fibrosis score <−1.455) was found in 41.4% of subjects, an intermediate probability of advanced liver fibrosis (NAFLD fibrosis score −1.455–0.676) was found in 48.9% of subjects, and a high probability of advanced liver fibrosis (NAFLD fibrosis score >0.676) was found in 9.6% of subjects. As expected by stratifying subjects according to the NAFLD fibrosis score, individuals classified as at high or intermediate probability of liver fibrosis were older (*P*<0.0001), had higher BMI (*P*<0.0001) and AST/ALT ratio (*P*<0.0001), lower platelet counts (*P*<0.0001) and albumin levels (*P*<0.0001), and were more likely to have elevated fasting glucose (*P*<0.0001) or IFG/IGT/type 2 diabetes (*P*<0.0001) as compared with those at low probability of liver fibrosis. A lower proportion of individuals classified as at high probability of liver fibrosis were current smokers (*P* = 0.04). Subjects classified as at high or intermediate probability of liver fibrosis were more likely to have metabolic syndrome (*P*<0.0001) as compared with those at low probability of liver fibrosis. A higher proportion of individuals classified as at high probability of liver fibrosis were treated with insulin (*P* = 0.01) (Table 1). A higher proportion of individuals classified as at high or intermediate probability of liver fibrosis were treated with statins (*P*<0.0001) (Table 1). In addition, significant differences between the three groups were observed with respect to anti-hypertensive treatments: a higher proportion of individuals classified as at high or intermediate probability of fibrosis were treated with ACE inhibitors, angiotensin receptor blockers and diuretics (*P*<0.0001) (Table 1).

**Table 1:** Anthropometric and biochemical characteristics of the study subjects stratified according to fibrosis risk score.

Variables	Whole study subjects	Low probability of fibrosis (< −1.455)	Intermediate probability of fibrosis (−1.455–0.676)	High probability of fibrosis (>0.676)	*P*
Gender (Male/Female)	319/251	126/110	162/117	31/24	0.56
Age *(yrs)*	54.1±13.5	47.0±12.4	57.7±11.1^d^	66.5±12.9^d^	<0.0001
BMI *(kg/m^2^)*	32.3±6.5	30.5±5.3	33.0±6.1^b^	36.9±9.3^d^	<0.0001
Waist circumference (c*m*)	107±14	102±12	109±13^d^	116±17^d^	<0.0001
Current smokers No (%)	102 (17.9%)	50 (21.2%)	48 (17.2%)	4 (7.3%)^a^	0.04
SBP *(mmHg)*	135±18	133±17	136±18	144±23^d^	<0.0001
DBP *(mmHg)*	82±11	82±10	82±11	81±12	0.82
Fasting glucose (*mg/dl*)	113±47	99±35	120±46^d^	143±67^d^	<0.0001
2-h post-load glucose (*mg/dl*)	137±45	123±39	148±48^d^	158±50^d^	<0.0001
Fasting insulin *(μU/ml)*	15±9	14±9	16±9^a^	18±10^a^	0.008
Total cholesterol (*mg/dl*)	197±40	204±37	193±40^b^	181±39^d^	<0.0001
HDL (*mg/dl*)	47±13	48±13	47±13	45±12	0.67
Triglycerides (*mg/dl*)	144±71	137±67	150±73	144±73	0.14
Uric acid (*mg/dl*)	5.5±1.4	5.3±1.3	5.6±1.4	6.3±1.8^d^	<0.0001
IGF-1 (*ng/ml*)	144±57	158±57	137±54^d^	117±46^d^	<0.0001
eGFR (ml/min/1.73m^2^)	93±25	102 ±27	87±21^d^	82±24^d^	<0.0001
CKD No, (%)	38 (6.7%)	6 (2.5%)	21 (7.5%)	11 (20.0%)	<0.0001
hsCRP (*mg/l*)	4.3±3.9	3.9±3.7	4.3±3.7	5.9±5.1^c^	0.003
Fibrinogen (*mg/dl*)	318±80	307±81	322±78^a^	343±85^b^	0.003
ALT (*UI/l*)	30±18	32±19	29±16^a^	23±16^c^	<0.0001
AST (*UI/l*)	25±14	23±11	25±12	30±25^b^	0.01
AST/ALT ratio	0.94±0.44	0.83±0.27	0.94±0.33^c^	1.41±0.96^d^	<0.0001
GGT (*UI/l*)	36±30	35±26	35±28	46±44	0.25
Platelet count (*x10^9^/l*)	249±71	288±76	230±49^d^	179±43^d^	<0.0001
Albumin (*g/dl*)	4.43±0.35	4.52±0.38	4.41±0.30^d^	4.14±0.29^d^	<0.0001
HOMA-IR index	4.2±3.7	3.5±2.9	4.5±2.9^b^	6.5±7.5^d^	<0.0001
NFG/IFG/IGT/T2DM (No)	220/63/112/175	158/18/29/31	58/39/69/113^d^	4/6/14/31^d^	<0.0001
Metabolic syndrome No (%)	392 (68.8%)	127 (53.8%)	219 (78.5%)^d^	46 (83.6%)^d^	<0.0001
Antidiabetic treatment (No)					
Diet/Oral hypoglycemic agents/Insulin	66/59/50	13/11/7	45/42/26	8/6/17	0.01
Therapy with statins No (%)	144 (25.3%)	38 (16.1%)	82 (29.4%)^c^	24(43.6%)^d^	<0.0001
ACE inhibitor therapy, No (*%*)	156 (27.4%)	53 (22.5%)	80 (28.7%)^b^	23(41.8%)^d^	<0.0001
Angiotensin receptor blocker therapy, No (*%*)	132 (23.2%)	38(16.1%)	77 (27.6%)^d^	17 (30.9%)^d^	<0.0001
Calcium channel blockers, No (*%*)	117 (20.5%)	46 (19.5%)	56 (20.1%)	15(27.3%)	0.42
Diuretics, No (*%*)	143 (25.1%)	30 (12.7%)	84 (30.1%)^d^	29 (52.7%)^d^	<0.0001

Data are means ± SD. Insulin, triglyceride, hsCRP, and GGT levels were log transformed for statistical analysis, but values in the table represent a back transformation to the original scale. Categorical variables were compared by χ^2^ test. *P* values refer to results after analyses with adjustment for gender. M =  male; F =  female; BMI =  body mass index; SBP =  systolic blood pressure; DBP =  diastolic blood pressure; HDL =  high-density lipoprotein; hsCRP =  high sensitivity C-reactive protein; ALT =  alanine aminotransferase; AST =  aspartate aminotransferase; GGT =  gamma-glutamyltransferase; HOMA-IR =  homeostasis model assessment index of insulin resistance; IGF-1 =  insulin-like growth factor-1; eGFR =  estimated glomerular filtration rate; CKD =  chronic kidney disease; ACE =  angiotensin-converting-enzyme; NFG =  normal glucose tolerance; IFG =  impaired fasting glucose; IGT =  impaired glucose tolerance; T2DM =  type 2 diabetes.

a *P*<0.05 vs. Low risk of fibrosis group.

b *P*<0.01 vs. Low risk of fibrosis group.

c *P*<0.001 vs. Low risk of fibrosis group

d *P*<0.0001 vs. Low risk of fibrosis group.

As compared with individuals at low probability of liver fibrosis, both individuals at high probability of fibrosis and individuals at intermediate probability of fibrosis showed an unfavorable cardio-metabolic risk profile having significantly higher values of waist circumference, insulin resistance, as assessed by the HOMA-IR index, hsCRP, fibrinogen, serum uric acid as well as lower levels of IGF-1 (Table 1). In addition individuals at high probability of fibrosis had higher systolic blood pressure values.

As compared with individuals at low probability of liver fibrosis, both individuals at high and those at intermediate probability of fibrosis exhibited lower value of eGFR (*P*<0.0001) (Table 1). These differences remained statistically significant after adjustment for smoking habit, glucose tolerance status, HOMA-IR index, diagnosis of metabolic syndrome, statin therapy, medications for diabetes, and anti-hypertensive treatments in addition to gender using a general linear model (*P* = 0.001) (Table 2). Furthermore, the differences remained statistically significant after adjustment for individual components of the metabolic syndrome including waist circumference, blood pressure, HDL, triglycerides, and glucose values in addition to gender (*P*<0.0001) (Table 3).

**Table 2:** General linear model with eGFR as the dependent variable.

Variables	F	*P*
Gender	1.62	0.20
Current smokers No (%)	0.19	0.66
HOMA-IR index	0.03	0.88
Glucose tolerance status	0.13	0.72
Metabolic syndrome diagnosis	0.57	0.45
Antidiabetic treatment	0.42	0.51
Therapy with statins	4.34	0.03
ACE inhibitor therapy	15.2	0.0001
Angiotensin receptor blocker therapy	1.97	0.16
Calcium channel blockers	0.02	0.96
Diuretics	10.6	0.001
Fibrosis risk score	6.99	0.001

eGFR =  estimated glomerular filtration rate; HOMA-IR =  homeostasis model assessment index of insulin resistance; ACE =  angiotensin-converting-enzyme.

**Table 3:** General linear model with eGFR as the dependent variable.

Variables	F	*P*
Gender	0.50	0.47
Waist circumference	16.58	0.0001
Fasting glucose	0.57	0.45
HDL	0.70	0.40
Triglycerides	4.20	0.04
Systolic blood pressure	15.57	0.0001
Diastolic blood pressure	7.49	0.006
Fibrosis risk score	17.96	0.0001

eGFR =  estimated glomerular filtration rate; HDL =  high-density lipoprotein.

When the analysis was restricted to the 175 subjects with IFG or IGT, both individuals at high and those at intermediate probability of fibrosis exhibited lower value of eGFR (95±19 and 100±26 ml/min/1.73 m^2^, respectively; *P* = 0.03) as compared with individuals at low probability of liver fibrosis (eGFR  = 110±25 ml/min/1.73 m^2^). Accordingly, when the analysis was restricted to the 175 subjects with type 2 diabetes, both individuals at high and those at intermediate probability of fibrosis exhibited lower value of eGFR (81±33 and 89±26 ml/min/1.73 m^2^, respectively; *P* = 0.001) as compared with individuals at low probability of liver fibrosis (eGFR  = 109±42 ml/min/1.73 m^2^).

Of the 570 subjects examined, 38 (6.7%) had CKD defined as eGFR <60 ml/min/1.73 m^2^. A logistic regression model adjusted for gender, age, and BMI was used to compare the risk of individuals at high and at intermediate probability of fibrosis to have CKD as compared with individuals at low probability of fibrosis (the reference category). Individuals at high probability of fibrosis had a 5.1-fold increased risk of having CKD (OR 5.13, 95%CI 1.13–23.28; *P* = 0.03) and individuals at intermediate probability of fibrosis had a 3.0-fold increased risk of having CKD (OR 3.01, 95%CI 0.87–10.32; *P* = 0.07) as compared with individuals at low probability of fibrosis. After adjustment for glucose tolerance status, statin therapy, and anti-hypertensive treatment in addition to gender, individuals at high probability of fibrosis had a 3.9-fold increased risk of having CKD (OR 3.94, 95%CI 1.11–14.05; *P* = 0.03) as compared with individuals at low probability of fibrosis (Table 4). Increased risk of CKD was also independently associated with glucose tolerance status (*P* = 0.03), and anti-hypertensive treatment (*P* = 0.002) (Table 4).

**Table 4:** Logistic regression analyses adjusted for gender of the association between study group subjects and CKD.

		CKD	
Variables	OR	95%CI	*P*
Individuals at low probability of fibrosis (reference category)	1	—	—
Individuals at high probability of fibrosis	3.94	1.11–14.05	0.03
Glucose tolerance status	1.33	1.02–1.75	0.03
Statin therapy	1.09	0.51–2.32	0.80
Anti-hypertensive treatment	1.04	1.01–1.06	0.002

CKD =  chronic kidney disease.

## Discussion

It is increasingly recognized that both NAFLD and CKD are associated with a clustering of traditional and non-traditional cardio-metabolic risk factors, and predict the development of cardiovascular diseases [1]–[6], [10], [12]–[26]. There is also evidence supporting the notion that adverse clinical outcomes are more frequent in patients with NASH rather than in individuals with simple liver steatosis [12]–[14], [17], [28], [29], thus emphasizing the importance to assess more advanced form of NAFLD in individuals affected by liver steatosis. Several noninvasive scoring indexes combining clinical and laboratory variables have been developed in order to identify advanced fibrosis in individuals with NAFLD [33]–[37]. Employing one of these liver fibrosis scores, it has been shown that advanced fibrosis is associated with increased risk of cardiovascular mortality in individuals with NAFLD [24], [25]. These findings coupled with the accessibility of a carefully characterized cohort of adult subjects have provided the rationale for investigating the relationship between advanced liver fibrosis, as determined by the NAFLD fibrosis score [34], in subjects with ultrasonography-diagnosed NAFLD and CKD. In this cross-sectional study, we report that individuals with high or intermediate probability of advanced liver fibrosis have lower eGFR as compared with individuals at low probability of liver fibrosis. These associations did not change after adjusting for several potential confounders including glucose tolerance status, diagnosis of metabolic syndrome or its individual components, treatments for dyslipidemia or hypertension. Accordingly, individuals with high probability of advanced liver fibrosis showed a 5.1-fold increased risk of having CKD as compared with individuals at low probability of fibrosis after adjustment for gender, age, and BMI. These data are consistent with those of two previous studies showing that CKD is associated with the severity of liver histopathology in adult individuals with biopsy-proven NAFLD [28], [29].

The underlying mechanism(s) by which NAFLD/NASH may contribute to kidney dysfunction are still unsettled. The most obvious explanation is that classical and non-classical cardio-metabolic risk factors, such as abdominal obesity, impaired glucose homeostasis/diabetes, hypertension, dyslipidemia, insulin resistance, metabolic syndrome, elevated serum uric acid, plasma inflammatory and hemostatic factors, all of which are associated with NAFLD/NASH may be also important risk factors for the development of CKD. Accordingly, we found that individuals with high or intermediate probability of advanced liver fibrosis have an unfavorable cardio-vascular risk profile characterized by an increase in visceral adiposity, insulin resistance, inflammatory and pro-coagulant biomarkers such as hsCRP, and fibrinogen. However, the strong relationships between these cardio-metabolic risk factors, NAFLD/NASH, and renal dysfunction make it extremely difficult to determine the precise cause-effect relationship between the two disorders. A potential plausible candidate linking NAFLD/NASH and renal dysfunction merits a comment. A number of evidences suggest that IGF-1 has effects on glomerular hemodynamics by enhancing both renal plasma flow and GFR [19], [45]–[47]. IGF-1 induces NO production in human umbilical vein endothelial cells, an effect that is abolished by a neutralizing IGF-1 receptor antibody [45], and renal vasodilation induced by IGF-1 is completely inhibited by an inhibitor of nitric oxide biosynthesis [46]. Studies in humans have shown that plasma IGF-1 levels are associated with GFR [19], and intravenous infusion of rhIGF-1 increases renal plasma flow and GFR in healthy subjects [47]. We found that individuals with high or intermediate probability of advanced liver fibrosis have lower circulating IGF-1 levels as compared with individuals at low probability of liver fibrosis. These findings are consistent with previous studies showing that plasma IGF-1 concentration is a determinant of eGFR in hypertensive individuals [19], and suggest that lower amounts of circulating IGF-1 associated with NAFLD/NASH [20], [21] could contribute to the reduced eGFR observed in individuals with high or intermediate probability of advanced liver fibrosis.

Several strengths and potential limitations of our study deserve comment. The major strengths of the study include the relatively large sample size with detailed anthropometric, clinical, and cardio-metabolic variables, the inclusion of both sexes, the ultrasound diagnosis of NAFLD performed by an experienced examiner who was blinded to the subjects' clinical and biochemical data, the use of the CKD-EPI equation to estimate GFR, which is more accurate than the Modification of Diet in Renal Disease (MDRD) study equation to estimate renal function in obese subjects characterized by a higher GFR, the use of restrictive *post hoc* Bonferroni test to correct for multiple comparisons, and the exclusion of confounding conditions characterized by elevation in liver biomarkers such as heavy drinking, positivity for antibodies to HCV or HBsAg and cirrhosis (thus excluding patients with hepatorenal syndrome).

Nevertheless, the present study has certain limitations that require consideration. First, only serum creatinine levels and estimated GFR were available, introducing inaccuracy into estimates of GFR and, potentially, a misclassification of subjects with impaired kidney function. Although gold standard methods to measure GFR (isotope clearance measurements) may provide a more sensitive estimate of renal function, they are time-consuming and expensive procedures which are not feasible in large-scale studies. However, estimated GFR based on serum creatinine facilitates the detection, evaluation, and management of CKD, and many organizations such as the National Kidney Foundation recommend the use of prediction equations for the evaluation of kidney function in epidemiologic studies and in clinical practice. Thus, our findings may be applicable to public health practice settings. Second, the diagnosis of NAFLD was based on ultrasonography rather than on invasive methods such as percutaneous liver biopsy. Although ultrasonography is the common method of diagnosing for hepatic steatosis in clinical practice, its sensitivity is suboptimal when hepatic fat infiltration of the liver is <30%. However, participants to our study had normal or only mildly elevated serum liver enzymes and, therefore, liver biopsy may be impractical for most of them. Additionally, all laboratory variables, including plasma glucose during OGTT were measured once, and small changes in the variables would therefore be expected if the same measurements were repeated on a different day. Although such an approach is common in clinical practice and in large epidemiologic studies, these assays are subject to intra-individual variability, and, therefore, some inaccuracy in the classification of subjects into glucose tolerance categories might have occurred. Furthermore, the information on alcohol intake was assessed by self-reported questionnaire, thus the real daily alcohol consumption may have been undervalued. Besides, our cohort includes outpatients recruited at a referral university hospital, representing subjects at increased risk for cardio-vascular disease, and, therefore, the present results may not be extendible to the general population. Moreover, all participants to the present study were White, and whether these findings also can be extended to other ethnic groups remains to be established. Finally, because of the cross-sectional design of this study, the present findings reflect only an association with prevalent and not incident kidney dysfunction, and therefore no definitive cause and effect relationship can be inferred.

The present cross-sectional findings may have both clinical and public health implications. Impaired renal function and NAFLD/NASH are two worldwide health problems due their devastating adverse outcomes, including end-stage renal disease, cirrhosis, hepatocellular carcinoma, and increased cardiovascular morbidity and mortality. Therefore, it appears necessary to identify those individuals who are at greatest risk for hepatic and renal diseases. The use of noninvasive scoring indexes for the prediction of fibrosis in subjects with NAFLD may be useful to identify those patients who need more stringent clinical surveillance aimed at preventing development or progression of both liver and renal complications.

## References

[1] Go AS, Chertow GM, Fan D, McCulloch CE, Hsu CY (2004) Chronic kidney disease and the risks of death, cardiovascular events, and hospitalization. N Engl J Med 351: 1296–1305. 15385656 10.1056/NEJMoa041031

[2] Weiner DE, Tighiouart H, Amin MG, Stark PC, MacLeod B, et al (2004) Chronic kidney disease as a risk factor for cardiovascular disease and all-cause mortality: a pooled analysis of community-based studies. J Am Soc Nephrol 15: 1307–1315. 15100371 10.1097/01.asn.0000123691.46138.e2

[3] Perticone F, Sciacqua A, Maio R, Perticone M, Laino I, et al (2009) Renal function predicts cardiovascular outcomes in southern Italian postmenopausal women. Eur J Cardiovasc Prev Rehabil 16: 481–486. 19531955 10.1097/HJR.0b013e32832b8d87

[4] Fox CS, Larson MG, Leip EP, Culleton B, Wilson PW, et al (2004) Predictors of new-onset kidney disease in a community-based population. JAMA 291: 844–850. 14970063 10.1001/jama.291.7.844

[5] Chen J, Muntner P, Hamm LL, Jones DW, Batuman V, et al (2004) The metabolic syndrome and chronic kidney disease in US adults. Ann Intern Med 140: 167–174. 14757614 10.7326/0003-4819-140-3-200402030-00007

[6] Sesti G, Succurro E, Arturi F, Andreozzi F, Laino I, et al (2011) IGF-1 levels link estimated glomerular filtration rate to insulin resistance in obesity: A study in obese, but metabolically healthy, subjects and obese, insulin-resistant subjects. Nutr Metab Cardiovasc Dis 21: 485–491. 20685093 10.1016/j.numecd.2010.02.008

[7] Browning JD, Szczepaniak LS, Dobbins R, Nuremberg P, Horton JD, et al (2004) Prevalence of hepatic steatosis in an urban population in the United States: impact of ethnicity. Hepatology 40: 1387–95. 15565570 10.1002/hep.20466

[8] Angulo P (2002) Nonalcoholic fatty liver disease. N Engl J Med 346: 1221–1231. 11961152 10.1056/NEJMra011775

[9] Petta S, Muratore C, Craxi A (2009) Nonalcoholic fatty liver disease pathogenesis: the present and the future. Dig Liver Dis 41: 615–25. 19223251 10.1016/j.dld.2009.01.004

[10] Bugianesi E, Leone N, Vanni E, Marchesini G, Brunello F, et al (2002) Expanding the natural history of nonalcoholic steatohepatitis: from cryptogenic cirrhosis to hepatocellular carcinoma. Gastroenterology 123: 134–140. 12105842 10.1053/gast.2002.34168

[11] Adams LA, Lymp JF, St Sauver J, Sanderson SO, Lindor KD, et al (2005) The natural history of nonalcoholic fatty liver disease: a population-based cohort study. Gastroenterology 129: 113–121. 16012941 10.1053/j.gastro.2005.04.014

[12] Ekstedt M, Franzen LE, Mathiesen UL, Thorelius L, Holmqvist M, et al (2006) Long-term follow-up of patients with NAFLD and elevated liver enzymes. Hepatology 44: 865–873. 17006923 10.1002/hep.21327

[13] Rafiq N, Bai C, Fang Y, Srishord M, McCullough A, et al (2009) Long-term follow-up of patients with nonalcoholic fatty liver. Clin Gastroenterol Hepatol 7: 234–238. 19049831 10.1016/j.cgh.2008.11.005

[14] Marchesini G, Brizi M, Bianchi G, Tomassetti S, Bugianesi E, et al (2001) Nonalcoholic fatty liver disease: a feature of the metabolic syndrome. Diabetes 50: 1844–1850. 11473047 10.2337/diabetes.50.8.1844

[15] Stefan N, Kantartzis K, Haring HU (2008) Causes and metabolic consequences of fatty liver. Endocr Rev 29: 939–960. 18723451 10.1210/er.2008-0009

[16] Stepanova M, Younossi ZM (2012) Independent association between nonalcoholic fatty liver disease and cardiovascular disease in the US population. Clin Gastroenterol Hepatol 10: 646–50. 22245962 10.1016/j.cgh.2011.12.039

[17] Targher G, Day CP, Bonora E (2010) Risk of cardiovascular disease in patients with nonalcoholic fatty liver disease. N Engl J Med 363: 1341–1350. 20879883 10.1056/NEJMra0912063

[18] Perticone F, Maio R, Sciacqua A, Perticone M, Laino I, et al (2009) Insulin-like growth factor-1 and glomerular filtration rate in hypertensive patients. J Hypertens 27: 613–617. 19330922 10.1097/hjh.0b013e32831fda24

[19] Petta S, Cammà C, Cabibi D, Di Marco V, Craxì A (2011) Hyperuricemia is associated with histological liver damage in patients with non-alcoholic fatty liver disease. Aliment Pharmacol Ther 34: 757–66. 21790685 10.1111/j.1365-2036.2011.04788.x

[20] Arturi F, Succurro E, Procopio C, Pedace E, Mannino GC, et al (2011) Nonalcoholic fatty liver disease is associated with low circulating levels of insulin-like growth factor-I. J Clin Endocrinol Metab 96: E1640–E1644. 21816784 10.1210/jc.2011-1227

[21] Hribal ML, Procopio T, Petta S, Sciacqua A, Grimaudo S, et al (2013) Insulin-Like Growth Factor-1, inflammatory proteins, and fibrosis in subjects with nonalcoholic fatty liver disease. J Clin Endocrinol Metab 98: E304–E308. 23316084 10.1210/jc.2012-3290

[22] Sciacqua A, Perticone M, Miceli S, Laino I, Tassone EJ, et al (2011) Endothelial dysfunction and non-alcoholic liver steatosis in hypertensive patients. Nutr Metab Cardiovasc Dis 21: 485–491. 20227260 10.1016/j.numecd.2009.11.015

[23] Perticone F, Maio R, Perticone M, Sciacqua A, Shehaj E, et al (2010) Endothelial dysfunction and subsequent decline in glomerular filtration rate in hypertensive patients. Circulation 122: 379–384. 20625109 10.1161/CIRCULATIONAHA.110.940932

[24] Targher G, Chonchol M, Miele L, Zoppini G, Pichiri I, et al (2009) Nonalcoholic fatty liver disease as a contributor to hypercoagulation and thrombophilia in the metabolic syndrome. Semin Thromb Hemost 35: 277–287. 19452403 10.1055/s-0029-1222606

[25] Weiner DE, Tighiouart H, Elsayed EF, Griffith JL, Salem DN, et al (2008) The relationship between nontraditional risk factors and outcomes in individuals with stage 3 to 4 CKD. Am J Kidney Dis 51: 212–223. 18215699 10.1053/j.ajkd.2007.10.035 PMC4083633

[26] Targher G, Chonchol M, Zoppini G, Abaterusso C, Bonora E (2011) Risk of chronic kidney disease in patients with non-alcoholic fatty liver disease: is there a link? J Hepatol 54: 1020–9. 21145850 10.1016/j.jhep.2010.11.007

[27] Targher G, Bertolini L, Rodella S, Zoppini G, Lippi G, et al (2008) Nonalcoholic fatty liver disease is independently associated with an increased prevalence of chronic kidney disease and proliferative/laser-treated retinopathy in type 2 diabetic patients. Diabetologia 51: 444–450. 18058083 10.1007/s00125-007-0897-4

[28] Targher G, Chonchol M, Bertolini L, Rodella S, Lippi G, et al (2010) Relationship between kidney function and liver histology in subjects with nonalcoholic steatohepatitis. Clin J Am Soc Nephrol 5: 2166–2171. 20724519 10.2215/CJN.05050610 PMC2994076

[29] Yasui K, Sumida Y, Mori Y, Mitsuyoshi H, Minami M, et al (2011) Nonalcoholic steatohepatitis and increased risk of chronic kidney disease. Metabolism 60: 735–9. 20817213 10.1016/j.metabol.2010.07.022

[30] Chang Y, Ryu S, Sung E, Woo HY, Oh E, et al (2008) Nonalcoholic fatty liver disease predicts chronic kidney disease in nonhypertensive and nondiabetic Korean men. Metabolism 57: 569–576. 18328362 10.1016/j.metabol.2007.11.022

[31] Targher G, Chonchol M, Bertolini L, Rodella S, Zenari L, et al (2008) Increased risk of CKD among type 2 diabetics with nonalcoholic fatty liver disease. J Am Soc Nephrol 19: 1564–1570. 18385424 10.1681/ASN.2007101155 PMC2488256

[32] Myers RP, Fong A, Shaheen AA (2008) Utilization rates, complications and costs of percutaneous liver biopsy: a population-based study including 4275 biopsies. Liver Int 28: 705–12. 18433397 10.1111/j.1478-3231.2008.01691.x

[33] Wai CT, Greenson JK, Fontana RJ, Kalbfleisch JD, Marrero JA, et al (2003) A simple noninvasive index can predict both significant fibrosis and cirrhosis in patients with chronic hepatitis C. Hepatology 38: 518–26. 12883497 10.1053/jhep.2003.50346

[34] Angulo P, Hui JM, Marchesini G, Bugianesi E, George J, et al (2007) The NAFLD fibrosis score: a noninvasive system that identifies liver fibrosis in patients with NAFLD. Hepatology 45: 846–854. 17393509 10.1002/hep.21496

[35] Sterling RK, Lissen E, Clumeck N, Sola R, Correa MC, et al (2006) Development of a simple noninvasive index to predict significant fibrosis in patients with HIV/HCV coinfection. Hepatology 43: 1317–1325. 16729309 10.1002/hep.21178

[36] Harrison SA, Oliver D, Arnold HL, Gogia S, Neuschwander-Tetri BA (2008) Development and validation of a simple NAFLD clinical scoring system for identifying patients without advanced disease. Gut 57: 1441–1447. 18390575 10.1136/gut.2007.146019

[37] Demir M, Lang S, Schlattjan M, Drebber U, Wedemeyer I, et al (2013) NIKEI: a new inexpensive and non-invasive scoring system to exclude advanced fibrosis in patients with NAFLD. PLoS One 8: e58360. 23555578 10.1371/journal.pone.0058360 PMC3608644

[38] Treeprasertsuk S, Björnsson E, Enders F, Suwanwalaikorn S, Lindor KD (2013) NAFLD fibrosis score: a prognostic predictor for mortality and liver complications among NAFLD patients. World J Gastroenterol 19: 1219–29. 23482703 10.3748/wjg.v19.i8.1219 PMC3587478

[39] Kim D, Kim WR, Kim HJ, Therneau TM (2013) Association between noninvasive fibrosis markers and mortality among adults with nonalcoholic fatty liver disease in the United States. Hepatology 57: 1357–65. 23175136 10.1002/hep.26156 PMC3622816

[40] Succurro E, Arturi F, Grembiale A, Iorio F, Fiorentino TV, et al (2011) One-hour post-load plasma glucose levels are associated with elevated liver enzymes. Nutr Metab Cardiovasc Dis 21: 713–718. 21764272 10.1016/j.numecd.2011.02.002

[41] The Expert Committee on the Diagnosis and Classification of Diabetes Mellitus (2013) Follow-up report on the diagnosis of diabetes mellitus. Diabetes Care 26: 3160–3167. 10.2337/diacare.26.11.3160 14578255

[42] Matthews DR, Hosker JP, Rudenski AS, Naylor BA, Treacher DF, et al (1985) Homeostasis model assessment: insulin resistance and beta-cell function from fasting plasma glucose and insulin concentrations in man. Diabetologia 28: 412–419. 3899825 10.1007/BF00280883

[43] Levey AS, Stevens LA, Schmid CH, Zhang YL, Castro AF 3rd, et al (2009) CKD-EPI (Chronic Kidney Disease Epidemiology Collaboration). A new equation to estimate glomerular filtration rate. Ann Intern Med 150: 604–612. 19414839 10.7326/0003-4819-150-9-200905050-00006 PMC2763564

[44] Alberti KG, Eckel RH, Grundy SM, Zimmet PZ, Cleeman JI, et al (2009) Harmonizing the metabolic syndrome: a joint interim statement of the International Diabetes Federation Task Force on Epidemiology and Prevention; National Heart, Lung, and Blood Institute; American Heart Association; World Heart Federation; International Atherosclerosis Society; and International Association for the Study of Obesity. Circulation120: 1640–1645. 10.1161/CIRCULATIONAHA.109.192644 19805654

[45] Tsukahara H, Gordienko DV, Tonshoff B, Gelato MC, Goligorsky MS (1994) Direct demonstration of insulin-like growth factor-I-induced nitric oxide production by endothelial cells. Kidney Int 45: 598–604. 7513035 10.1038/ki.1994.78

[46] Haylor J, Singh I, el Nahas AM (1991) Nitric oxide synthesis inhibitor prevents vasodilation by insulin-like growth factor I. Kidney Int 39: 333–335. 2002647 10.1038/ki.1991.42

[47] Giordano M, DeFronzo RA (1995) Acute effect of human recombinant insulin-like growth factor I on renal function in humans. Nephron 71: 10–15. 8538827 10.1159/000188667

